# Tobacco and household expenditure in Egypt: insights into socioeconomic inequalities and spending profiles from the Household Income, Expenditure and Consumption Survey

**DOI:** 10.1186/s12889-025-21676-w

**Published:** 2025-02-13

**Authors:** Aya Mostafa, Rasha Saad Hussein

**Affiliations:** Department of Community, Environmental, and Occupational Medicine, Faculty of Medicine, Ain Shams University, Cairo, Egypt

**Keywords:** Tobacco expenditure, Income quintiles, Poverty, Inequality, Household, Health policy, Public health, Cigarettes, Waterpipe, Egypt

## Abstract

**Background:**

Tobacco use deepens poverty. Egypt, a lower-middle income country, is one of the few countries worldwide where tobacco use is rising. However, no published study examined the adverse impacts of tobacco on the Egyptian household welfare, specifically after the first Egyptian Pound (EGP) devaluation by 80% in 2016. To address this gap and inform tobacco taxation policymaking, we aimed to provide evidence characterizing national household tobacco expenditure in Egypt.

**Methods:**

We conducted a secondary analysis of the 2017/2018 Household Income, Expenditure and Consumption Survey, focusing on the most used tobacco products in Egypt: cigarette and waterpipe tobacco. We identified the proportion and background characteristics of cigarette and waterpipe tobacco smoker households. We calculated household tobacco expenditure share as a proportion of total household expenditure. We compared mean household expenditure shares of 12 expenditure groups among smoker and nonsmoker households and examined the differences by income quintiles. We determined the factors associated with household tobacco expenditure. Descriptive, bivariable, and multivariable analyses were performed.

**Results:**

Cigarette and waterpipe tobacco smokers were present in 41.1% and 7.0% of 12,845 households, respectively. Annual household expenditure on cigarettes (10.7%) was triple that of waterpipe tobacco (3.4%) (*p* < 0.001). Smoker households spent less than nonsmoker households on virtually all expenditure groups (*p* < 0.001). The poorest income quintile spent 11.1% of its total expenditure on tobacco (1.26 times higher than the richest, *p* = 0.006). More waterpipe tobacco than cigarette smoker households lived below the poverty line (40.6% versus 24.4%, *p* < 0.001). Cigarette smoker households spent less on food and housing but more on tobacco than waterpipe tobacco smoker households. The poorest cigarette and waterpipe tobacco smoker households spent 7.0-9.7 times as much on tobacco as on education. Common independent factors associated with higher household expenditure on cigarettes and waterpipe tobacco were urban residence (*p* = 0.011 and *p* = 0.015, respectively), and lower income (*p* < 0.001).

**Conclusion:**

In 2017/2018, one-tenth of Egyptian smoker household’s expenditure was allocated to tobacco, disproportionately concentrated among the poorest. Our results preliminarily indicate that tobacco expenditure is associated with potential compromises of varying extent in almost all other expenditures in smoker households. This baseline profiling of household tobacco expenditure can potentially inform an evidence-based tobacco taxation policy, supporting the reduction of tobacco-associated socioeconomic inequalities.

## Introduction

Tobacco hinders a country’s economic development [1]. Annually, 2.1% of Egypt’s gross domestic product (GDP) is lost due to tobacco-related morbidity and mortality, including 75% premature deaths [2]. Tobacco smoking does not only harm smokers, causing respiratory and cardiovascular diseases, cancers, and nicotine dependence, but its environmental smoke exposes people to dangerous toxicants [1]. Half of survey respondents reported household exposure to tobacco smoke in Egypt in the latest 2017 STEPwise Survey for Noncommunicable Diseases (STEPs) [3]. Moreover, tobacco use exacerbates poverty and deprives households from other necessary commodities [1, 4], as reported by studies in Africa [5], Asia [6], and Latin America [7]. The Updated Toolkit on Using Household Expenditure Surveys for Research in the Economics of Tobacco Control examined studies from China, India, South Africa, Cambodia, Turkey, Bangladesh, Chile, Mauritius, Vietnam, Kenya, Serbia, Indonesia, Montenegro, and an international study in 40 low- and middle-income countries [4]. All these studies commonly reported that spending on tobacco was associated with reduced spending on education, as well as other items that differed from one country to another, such as food, housing, clothing, and transportation [4]. These studies were conducted using different methods ranging from descriptive analyses to advanced econometric methods, and each method had its limitations [4]. Thus, the toolkit authors recommended in cases where advanced analysis methods are unavailable, that less sophisticated methods be implemented, such as a simple comparison of expenditure shares between tobacco spending and non-spending households on various commodities [4]. However, there are no published studies examining the adverse impacts of tobacco expenditure on the Egyptian household welfare.

**Fig. 1:**
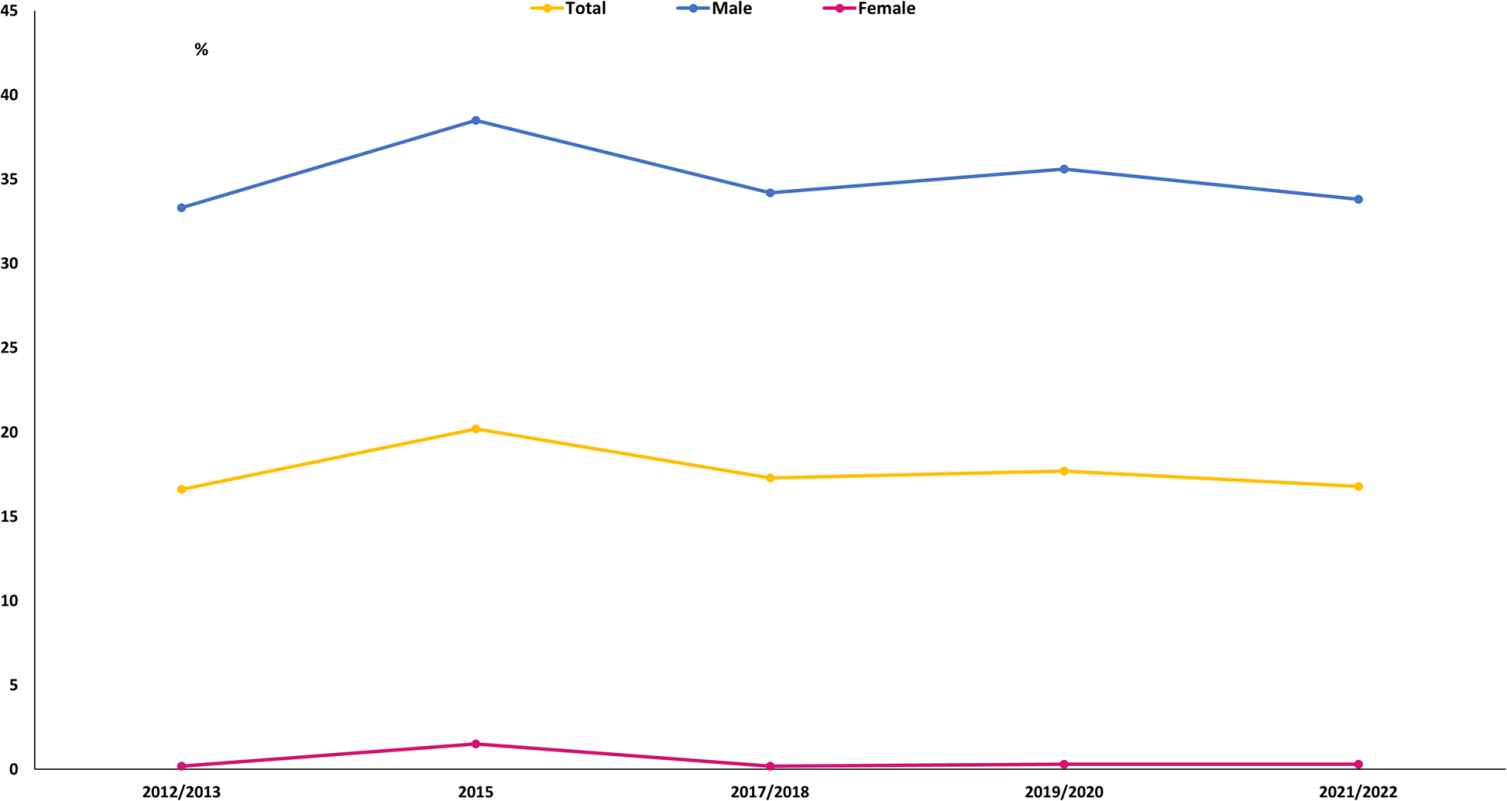
Prevalence of tobacco use. Household Income, Expenditure and Consumption Surveys (2012–2022), Egypt

Although Egypt has existing tobacco control interventions [8], the sub-optimal implementation of these policies failed to significantly reduce tobacco use in the last decade **(**Fig. 1**)**. In 2016/2017, smoking prevalence in Egyptian adolescents was alarmingly high (14.3%), reaching almost two-thirds of adult estimates (22.7%) [9]. Waterpipe tobacco is the second most used tobacco product in Egypt after cigarettes, and is twice as affordable [3, 8]. However, recent evidence on national household expenditure on waterpipe tobacco is lacking, last published in the 2009 Global Adult Tobacco Survey (GATS) [10]. Although the 2017 STEPs detailed various aspects of tobacco use; expenditure data were available only for cigarettes not waterpipe tobacco [3]. If made available together with proper characterization of household tobacco expenditure, simulations of health and financial benefits could be modelled to inform distributional effects of a uniform and comprehensive tobacco taxation policy.

**Fig. 2:**
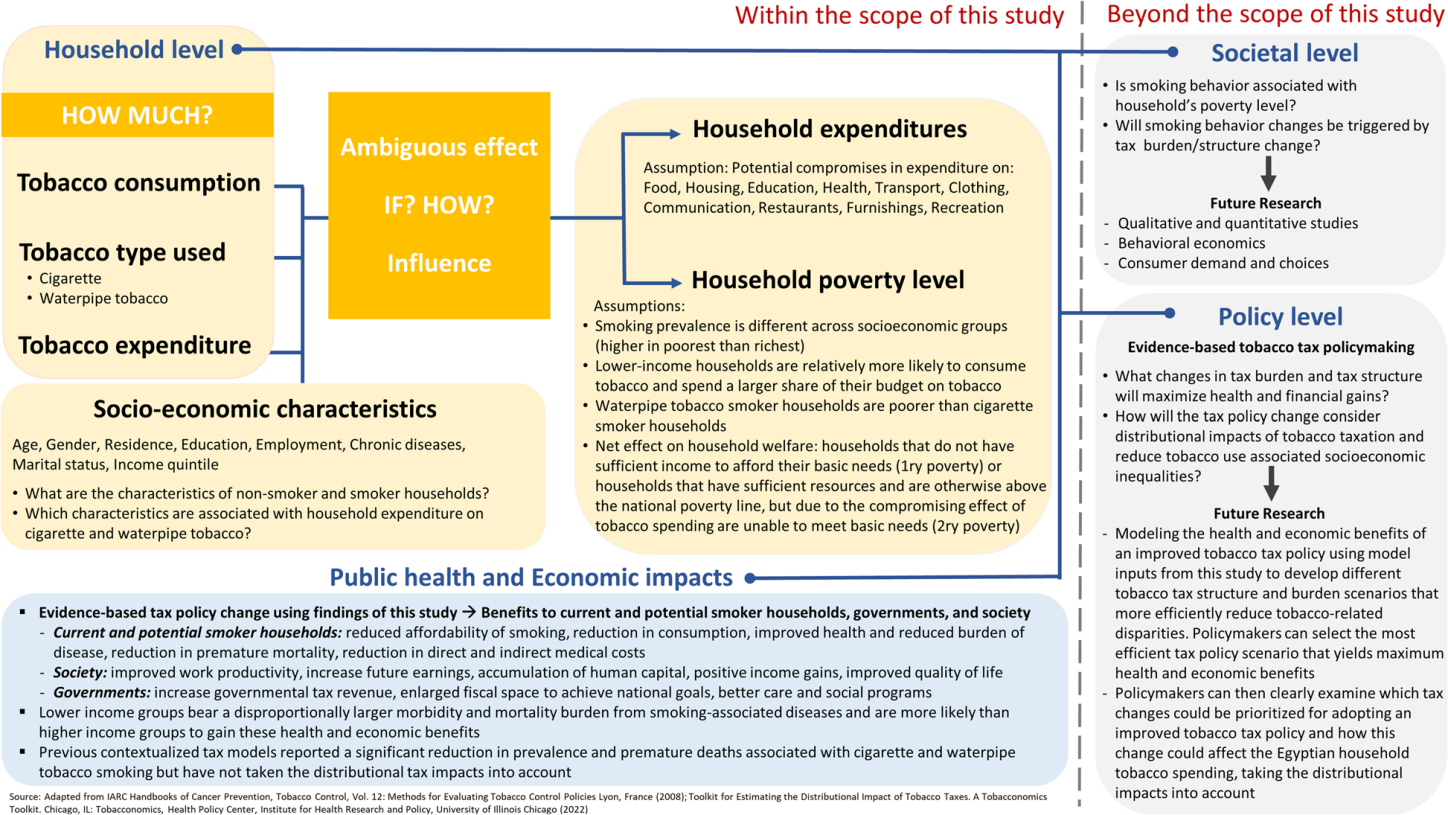
Theoretical framework of the study

Taxation is a cost-effective tobacco control policy tool that enables governments to reduce health burden and maximize financial revenues [1]; these gains benefit mostly the poorest 20% of the population [11]. Increased revenues from tobacco taxation will eventually create the fiscal space needed for Egypt to meet the 2030 Sustainable Development Goals [1]. The 2017/2018 national Household Income, Expenditure and Consumption Survey (HIECs) reported a 17% increase in poverty rate compared to 2015 [12]. In November 2016, the first devaluation of the Egyptian Pound (EGP) took place causing a 78% decline of its value to United States Dollar (USD) (1 USD = 15.8 EGP) post devaluation [13]. Inflation rates reached record numbers in mid-2017 (33%) [14] with increase in prices of all commodities, including tobacco. Based on previous literature [1, 4–7], we hypothesized that tobacco use is associated with the households’ level of poverty. Our theoretical framework is presented in Fig. 2. The analysis of HIECs 2017/2018 could provide baseline evidence on the Egyptian population’s consumption and expenditure behavior, investigating if and how expenditure on tobacco affects household spending on different expenditure groups.

To inform evidence-based tobacco taxation policymaking, we aimed in the analysis of HIECs 2017/2018 to: (a) identify the proportion of cigarette and waterpipe tobacco smoker households and their background characteristics; (b) calculate tobacco expenditure and its proportion of total household expenditure; (c) compare mean household expenditure shares between smoker and nonsmoker households, as well as among cigarette and waterpipe tobacco smoker households, and examine the differences by income quintile; and (d) determine the factors associated with household expenditure on cigarette and waterpipe tobacco.

## Methods

### Study design, sample, and tools

 This study is a secondary analysis of the nationwide household population-based cross-sectional survey, the 2017/2018 Household Income, Expenditure and Consumption Survey (HIECs) in Egypt. HIECs was conducted by the Egyptian Central Agency for Mobilization and Statistics (CAPMAS) [12]. HIECs 2017/2018 had a two-stage stratified cluster random sampling technique. The stratification in HIECs 2017/2018 was based on urban (45%) / rural (55%) residence and the cluster size was 20 households per enumeration area in all governorates [12]. The target sample size was 25,800 households. The overall response rate was 96.0%; 24,791 households in 27 governorates participated in the survey. The survey was conducted between October 2017 and September 2018. Data were collected from adults (≥ 18 years old) through household interviews using laptops. The survey questionnaire consisted of two main sections (Sect. 1: household data; Sect. 2: expenditure and consumption data for commodity and service groups) [12].

### Measures

For this analysis, CAPMAS randomly selected a sample of approximately 50% of the original HIECs 2017/2018 survey data and provided the author with a dataset including data for 12,485 households. The dataset included grouped raw data of selected items from the survey questionnaire (such as expenditure groups). On special request, CAPMAS provided some additional data on household tobacco use and expenditure. Data that were available for the heads of households were socio-demographic and background characteristics, including current smoking status. Data that were available at the household level were residence, annual household tobacco use and expenditure as well as annual household income and expenditure. No individual level data were available in these datasets.

### Sociodemographic and background characteristics

Sociodemographic and background data were available for heads of households only: age; gender (male/female); education (primary or less, intermediate, secondary, university or higher); employment (full-time, part-time, unemployed); marital status (married/unmarried); history of chronic diseases (yes/no); and type of chronic diseases relevant to tobacco use (hypertension, cardiovascular diseases, diabetes, respiratory diseases, tumors). The head of household reported the household residence as urban/rural.

### Tobacco use and expenditure

Heads of households were asked about their current tobacco smoking status (yes/no). The dataset did not include the current smoking status of other household members. Annual household use of and expenditure (in EGP) on smoked tobacco, i.e. cigarettes, waterpipe tobacco, and other smoked tobacco was reported by the heads of households; thus, this included expenditure for all household smokers not just the head of household. In this analysis, a smoker household was defined as whether the household had any household expenditure on tobacco — reflecting that there were one or more smokers (of any type of smoked tobacco) in the household, and a nonsmoker household was defined as the household not having any household expenditure on tobacco — reflecting that there were no smokers in the household. In Fig. 1, we present the prevalence of tobacco use that we obtained from previous HIECs surveys between 2012 and 2022. We used the chi-square goodness of fit test to detect any significant trend in the prevalence of tobacco use over that decade.

### Household income and expenditure

Expenditures in the dataset were reported by the head of household. HIECs provided annual grouped data for 12 main groups of household expenditure: (1) food and nonalcoholic beverages, (2) Housing, water, electricity, gas and other fuels “housing”, (3) Health, (4) Transport, (5) Clothing and footwear, (6) Alcoholic beverages, tobacco and narcotics “tobacco”, (7) Restaurants and hotels, (8) Education, (9) Furnishings, household equipment and maintenance, (10) Communication, 11) Recreation and culture, and 12) Miscellaneous goods and services [8]. Income was defined in the dataset as the net income of the family per year. The annual total household income and expenditure data were available in EGP. The mean household tobacco expenditure in all households, regardless of the household’s smoking status, was very close to that reported for the expenditure group “Alcoholic beverages, tobacco and narcotics”, indicating that the difference allocated for household expenditure on alcohol and narcotics is negligible. Therefore, we refer to this group as “tobacco” in the manuscript.

### Poverty

The percentage of households that were categorized into poor and not poor was available as a pre-calculated variable in the provided dataset. HIECs defines the poor as the population whose total consumption is less than the cost of the components of the national poverty line [12]. The national poverty line in 2017/2018 was 8827 EGP for one individual per year [12]. So, a family of 5 would need 44,136 EGP per year to be able to meet their basic needs. We used this variable only in describing the households **(**Table 1**)**, but we did not use it in further analysis **(**Tables 2, 3, 4, 5 and 6**)**; instead, we calculated income and expenditure quintiles as described under statistical analyses.

### Statistical analyses

To address the sample design of the HIECs 2017/2018, which is a two-stage stratified cluster random sampling technique, and to ensure the estimates derived from this analysis are representative, we applied the weight variable provided by CAPMAS in all analyses (the basic weight for each household in the sample is equal to the inverse of its selection probability, as described in the HIECs 2017/2018 manual) [12]. The unweighted and weighted estimates were very close. We reported the weighted numbers and estimates in all tables. We considered a p-value less than 0.05 statistically significant. We used MedCalc Software version 23.0.9 to estimate the p-value for some comparisons. SPSS version 25 was used for all analyses.

### Descriptive analysis

In the descriptive analyses, we calculated numbers and proportions for categorical variables. For continuous variables, such as tobacco expenditure and household expenditure and income, we calculated means, medians, and interquartile ranges (IQR). The annual total household income and expenditure were categorized into five quintiles after calculating the 20th, 40th, 60th, 80th, 100th percentiles. The 1st income quintile represents the poorest 20% and the 5th quintile represents the richest 20% of the sample. The proportion of smoking households was calculated as the number of households (may include one or more smokers in the household) that reported tobacco use and expenditure divided by the total households in the dataset. We calculated the household expenditure shares for the 12 main expenditure groups as a proportion of total household expenditure, by dividing each household’s expenditure on each group by the annual total household expenditure for that household, then we calculated and reported the mean of those expenditure shares and presented the shares in the text and tables as percentages by income quintile group. The proportion of tobacco expenditure in smoking households (for any smoking member in the household including the head) was calculated (by total and by tobacco type) following the same method used to calculate other household expenditure shares. The 95% confidence intervals (CI) are presented for these proportions.

### Bivariable analysis

In the bivariable analyses, we conducted independent samples t-tests to detect statistically significant differences in mean expenditure shares by nonsmoker and smoker households, and present these differences in the total sample and by income quintile [4, 5]. If the test for Equality of Variances was statistically significant, indicating unequal group variances in this population, the p-value of equal variances not assumed was reported. Also, we used chi-squared tests to examine the differences in background characteristics between smoker and nonsmoker households and to compare cigarette and waterpipe tobacco smoker households, each separately, to other households. We performed One-way Analysis of Variance (ANOVA) to compare differences in the mean expenditure shares of each of the 12 expenditure groups by income quintiles. If the Levene’s Test for Equality of Variances was statistically significant, indicating unequal group variances in this population, a correction using an adjustment to the degrees of freedom with the Welch-Satterthwaite method was done. If the F-value was significant, we used post hoc comparisons using Bonferroni correction for multiple comparisons to detect statistically significant differences mainly between the 1st and the 5th quintiles (the planned comparison).

### Multivariable analyses

In the multivariable analyses, we conducted two separate generalized linear (lognormal) models to determine factors associated with the annual household expenditure on cigarettes and waterpipe tobacco as the dependent variables (outcomes), which were entered into the model as continuous measures. The independent variables included in both models were (a) characteristics of the head of household: gender (male/female); age (< 50 years old/ ≥50 years old); education (primary or less, intermediate, secondary, university or higher); employment (full-time, part-time, unemployed); marital status (married/unmarried); history of chronic diseases (yes/no); as well as (b) characteristics of the household: residence (urban/rural); and income quintiles (1st poorest/2nd /3rd /4th /5th richest). Poverty was not included in the models due to its moderate multicollinearity [15] (VIF = 2.823) with income and expenditure quintiles. We report the β coefficient and standard error for all independent variables and the Wald chi-square value.

#### Ethical considerations

This study was reviewed and approved by the Research Ethics Committee, Faculty of Medicine, Ain Shams University (FMASU P01/2019). We performed secondary analyses of primary data collected from respondents who provided informed consents before completing the interviews in 2017/2018 HIECs [12].

## Results

### Sample background characteristics

More than a half of the households were rural. More than one-third (35.1%) of the heads of households were current tobacco smokers, almost a fifth of them were females and a similar proportion were unmarried, and a quarter of them were below the age of 40. Most of the heads of households had less than secondary education and almost one-third reported they were unemployed at the time of the survey. Less than one-third of the heads of households reported having chronic diseases, primarily, hypertension and diabetes. A quarter of the households in this sample fell below the poverty line **(**Table 1**).**

**Table 1:** Background characteristics of heads of households by smoking status and type (cigarette and waterpipe), HIECs 2017/2018, Egypt

Background characteristics of heads of households	Total		Nonsmoker households		Smoker households		*p*-value^a^	Cigarette smoker households		*p*-value^b^	Waterpipe tobacco smoker households		*p*-value ^c^
	*N* = 12,485	%	*n* = 6717	%	*n* = 5768	%		*n*= 5131	%		*n*= 872	%	
**Residence**													
Rural	6760	54.1	3554	52.9	3207	55.6	0.003	2709	52.8	0.011	675	77.5	< 0.001
Urban	5725	45.9	3164	47.1	2561	44.4		2422	47.2		196	22.5	
**Current smoker**													
No	8101	64.9	6717	100	1388	24.1	< 0.001	1260	24.6	< 0.001	156	17.9	< 0.001
Yes	4384	35.1	0	0	4380	75.9		3871	75.4		716	82.1	
**Gender**													
Male	10,183	81.6	4883	72.7	5300	91.9	< 0.001	4695	91.5	< 0.001	831	95.4	< 0.001
Female	2302	18.4	1834	27.3	467	8.1		437	8.5		40	4.6	
**Age**													
18–30	528	4.2	271	4.0	257	4.5	<0.001	244	4.7	<0.001	19	2.2	<0.001
31–40	2493	20.0	1254	18.7	1239	21.5		1144	22.3		131	15.0	
41–50	3205	25.7	1598	23.8	1607	27.9		1393	27.1		276	31.7	
51–60	2987	23.9	1499	22.3	1488	26.8		1324	25.8		235	26.9	
≥ 61	3272	26.2	2095	31.2	1177	20.4		1026	20.0		211	24.2	
**Education**													
Less than intermediate	6735	53.9	3325	49.5	3409	59.1	<0.001	2981	58.1	< 0.001	613	70.3	<0.001
Intermediate	3450	27.6	1853	27.6	1596	27.7		1447	28.2		190	21.8	
Secondary	472	3.8	283	4.2	189	3.3		175	3.4		23	2.7	
University or higher	1829	14.6	1255	18.7	573	9.9		529	10.3		46	5.3	
**Employment**													
Unemployed	3772	30.2	2512	37.4	1259	21.8	<0.001	1142	22.3	<0.001	163	18.7	< 0.001
Part time	1628	13.0	646	9.6	982	17.0		864	16.8		156	17.9	
Full time	7085	56.7	3559	53.0	3526	61.1		3126	60.9		552	63.4	
**Marital status**													
Not married	2422	19.4	1772	26.4	650	11.3	<0.001	600	11.7	<0.001	70	8.1	< 0.001
Married	10,063	80.6	4946	73.6	5118	88.7		4531	88.3		801	91.9	
**Chronic disease**													
No	8641	69.2	4312	64.2	4329	75.1	<0.001	3832	74.7	< 0.001	686	78.7	<0.001
Yes	3844	30.8	2405	35.8	1439	24.9		1300	25.3		185	21.3	
**Chronic disease type**													
Hypertension	2384	19.1	1603	23.9	781	13.5	< 0.001	702	13.7	< 0.001	109	12.5	< 0.001
Cardiovascular	789	6.3	503	7.5	287	5.0	< 0.001	264	5.2	< 0.001	25	2.9	< 0.001
Diabetes	1790	14.3	1101	16.4	689	11.9	< 0.001	630	12.3	< 0.001	83	9.5	< 0.001
Respiratory	432	3.5	252	3.7	180	3.1	0.055	162	3.2	0.122	25	2.9	0.320
Tumors	49	0.4	30	0.5	18	0.3	0.226	17	0.3	0.423	0	0	0.055
**Annual total household income quintiles**													
1st (poorest) (5802.0 to 33280.0 EGP)	2497	20.0	1663	24.8	834	14.5	<0.001	670	13.1	<0.001	199	22.8	0.004
2nd (33286.0 to 43739.0 EGP)	2497	20.0	1294	19.3	1203	20.9		1063	20.7		177	20.3	
3rd (43750.0 to 55035.0 EGP)	2498	20.0	1230	18.3	1267	22.0		1142	22.3		190	21.8	
4th (55055.0 to 72500.0 EGP)	2497	20.0	1212	18.0	1285	22.3		1174	22.9		172	19.8	
5th (richest) (72511.5 to 7480000.0 EGP)	2497	20.0	1318	19.6	1178	20.4		1082	21.1		134	15.4	
**Mean annual household income**	59614.0		59462.0		59790.9		0.853^d^	60456.3		0.014^e^	56159.2	0.312^f^	
**Median annual household income**	48960.0		47267.0		50896.4			51779.0			47155.0		
**Annual total household expenditure quintiles**													
1st (poorest) (7554.4 to 32245.4 EGP)	2497	20.0	1811	27.0	686	11.9	<0.001	531	10.4	<0.001	172	19.7	0.001
2nd (322250.6 to 41222.5 EGP)	2496	20.0	1316	19.6	1181	20.5		1041	20.3		192	22.1	
3rd (41341.0 to 50953.9 EGP)	2498	20.0	1179	17.5	1319	22.9		1178	23.0		207	23.7	
4th (50961.0 to 65110.8 EGP)	2498	20.0	1184	17.6	1314	22.8		1200	23.4		164	18.8	
5th (richest) (65119.0 to 1287371.5 EGP)	2496	20.0	1228	18.3	1268	22.0		1181	23.0		137	15.7	
**Mean annual household expenditure**	52782.4		50172.4		55822.0		< 0.001^d^	56793.2		< 0.001^e^	50362.1	0.031^f^	
**Median annual household expenditure**	45697.0		42868.5		48586.1			49453.0			43885.0		
**Median household size**	4.0		4.0		5.0			5.0			5.0		
**Household is under poverty line**													
No	9454	75.7	5201	77.4	4253	73.7	<0.001	3881	75.6	0.829	517	59.4	< 0.001
Yes	3031	24.3	1516	22.6	1515	26.3		1251	24.4		354	40.6	

a Chi-squared test between smoker and nonsmoker households

b Chi-squared test between cigarette smoker households and other households. The null hypothesis was there was no difference in background characteristics between cigarette smoker households and other households

c Chi-squared test between waterpipe tobacco smoker households and other households. The null hypothesis was there was no difference in background characteristics between waterpipe tobacco smoker households and other households

d Independent samples t-test between smoker and nonsmoker households

e Independent samples t-test between cigarette smoker households and other households, where mean income and expenditure are higher in cigarette smoker households than in other households

f Independent samples t-test between waterpipe tobacco smoker households and other households, where mean income and expenditure are lower in waterpipe tobacco smoker households than in other households

### Proportion of cigarette and waterpipe tobacco smoker households and their background characteristics

The background characteristics of nonsmoker and smoker households are reported in Table 1. Of the total households, 7.0% reported waterpipe tobacco smoking, while 41.1% reported cigarette smoking **(**Table 2**)**. A higher proportion of waterpipe tobacco than cigarette smoker households lived in rural areas (77.5% versus 52.8%, *p* < 0.001), and had heads who were male, older, employed, and had lower education levels, fewer chronic diseases, lower annual income and expenditure, and lived under the poverty line (40.6% versus 24.4%, *p* < 0.001) **(**Table 1**)**. The prevalence of tobacco use from different HIECs surveys conducted between 2012 and 2022 is presented in Fig. 1. There seems to be a slight inconsistent decline in the prevalence of tobacco use over the past decade, however, the decline was not statistically significant.

**Table 2:** Household tobacco use and annual household tobacco expenditure, HIECs 2017/2018, Egypt

Smoked tobacco product	Household tobacco use ^a^		Annual household tobacco expenditure ^b^		Proportion of annual total household expenditure ^c^
	*N* = 12,485	%	median	IQR	(95%CI)
**Any smoked tobacco**	5768	46.2	4704.0	2700.0–6300.0	10.2 (10.0, 10.4)
**Cigarettes**	5131	41.1	5238.0	3060.0–6660.0	10.7 (10.6, 10.9)
**Waterpipe tobacco**	872	7.0	2016.0	990.0–3960.0	3.4 (3.4, 3.8)
**Other smoked tobacco**	117	0.9	2970.0	1067.9–7143.6	3.5 (2.9, 4.1)

a Tobacco use categories are not mutually exclusive because respondents may use more than one product. As smoker households may have an overlap of the tobacco type smoked, the proportions do not add up to 100%

b Amount spent on only the specific type of tobacco as reported by head and individuals in the households

c Calculated by dividing the mean annual expenditure on the specific type of tobacco by the mean annual total household expenditure

### Tobacco expenditure and its proportion of total household expenditure

Among smoking households in HIECs 2017/2018, the median annual total household expenditure on any smoked tobacco was 4704.0 EGP (IQR 2700.0–6300.0) representing 10.2% (95%CI: 10.0, 10.4) of the annual total household expenditure. The median annual household expenditure on cigarettes was 5238.0 EGP (IQR 3060.0–6660.0) and on waterpipe tobacco was 2016.0 EGP (IQR 990.0–3960.0), representing 10.7% (95% CI: 10.6, 10.9) and 3.4% (95% CI: 3.4, 4.1) of the annual total household expenditure, respectively **(**Table 2**)**.

### Household expenditure shares by income quintile

Compared to the 5th (richest) income quintile, the mean expenditure share of the 1st (poorest) quintile was significantly higher on food and nonalcoholic beverages (10.4%) (*p* < 0.001) and housing (4.3%) (*p* < 0.001). The 5th (richest) quintile spent significantly more than the 1st (poorest) quintile on almost all other categories, specifically on education (4.2%) (*p* < 0.001) and transportation (3.5%) (*p* < 0.001) **(**Table 3**)**.

**Table 3:** Annual household expenditure shares by income quintiles, HIECs 2017/2018, Egypt

Expenditure share (%) ^a^	Income quintiles ^c^					Mean expenditure share ^d^(%)	Mean annual household expenditure (in EGP)
	1st (poorest)	2nd	3rd	4th	5th (richest)		
**Food and nonalcoholic beverages**	43.5	41.1	39.9	38.1	33.1	39.1	19052.3
**Housing**,** water**,** electricity**,** gas and other fuels**	21.9	19.7	18.4	17.2	17.6	18.9	9476.0
**Health**	10.0	8.7	8.9	9.5	10.2	9.5	5193.3
**Transport**	3.6	4.1	4.5	5.2	7.1	4.9	3209.2
**Clothing and footwear**	3.0	4.1	4.7	5.1	5.3	4.4	2498.6
**Alcoholic beverages, tobacco and narcotics**	3.7	5.3	5.2	5.2	4.2	4.7	2436.7 ^d^
**Restaurants and hotels**	3.7	4.0	4.2	4.3	4.4	4.1	2251.7
**Education**	1.2	2.2	3.3	3.8	5.4	3.2	2316.2
**Furnishings, household equipment and maintenance**^b^	3.8	3.8	3.6	3.7	3.6	3.7	1986.4
**Communication**	1.6	2.0	2.2	2.4	2.8	2.2	1216.1
**Recreation and culture**	0.8	1.2	1.3	1.5	2.4	1.4	1085.0
**Miscellaneous goods and services**	3.3	3.8	3.9	4.0	4.0	3.8	2060.7

a Differences in mean expenditure shares between all quintile groups (combined) are statistically significant at the 1% level (the p-value is < 0.001), ANOVA. Pairwise comparisons were done by independent samples t-test

b The difference is statistically significant at the 5% level (p-value = 0.016)

c The 1st income quintile represents the poorest 20% and the 5th quintile represents the richest 20% of the sample

d Mean in both smoker and nonsmoker households

### Differences in mean expenditure shares by smoker and nonsmoker and by cigarette and waterpipe tobacco smoker households

Smoker households spent less on all categories, except restaurants and hotels and miscellaneous goods and services. Differences were larger within the lower than within the higher income quintiles. The largest difference in mean expenditure shares between smoker and nonsmoker households was between the 1st (poorest) and 5th (richest) quintiles on housing, food and nonalcoholic beverages, and health (3.6%, 3.4%, 2.0%, respectively) (*p* < 0.001). For tobacco in smoker households, the highest mean tobacco expenditure share was in the 1st (11.1%) and 2nd (10.9%) quintiles, with lower expenditure shares in the 3rd (10.3%) and 4th (10.1%) quintiles. The 5th quintile was the least spending on this category (8.8%) **(**Table 4**)**.

**Table 4:** Average annual household expenditure share and difference in mean expenditure share (%) between nonsmoker and smoker households by income quintile, HIECs 2017/2018, Egypt

Expenditure share (%)	Income quintiles					Total
	1st (poorest)	2nd	3rd	4th	5th (richest)	
Food and nonalcoholic beverages						
Nonsmoker	44.9	42.8	41.4	39.7	33.5	40.7
Smoker	40.7	39.1	38.3	36.6	32.7	37.3
Difference in mean expenditure share^a^	4.3^b^	3.7^b^	3.1^b^	3.1^b^	0.8^c^	3.4^b^
**Housing**,** water**,** electricity**,** gas and other fuels**						
Nonsmoker	23.4	21.0	19.6	18.6	19.4	20.6
Smoker	19.0	18.2	17.2	15.9	15.5	17.0
* Difference in mean expenditure share*^a^	4.4^b^	2.8^b^	2.5^b^	2.7^b^	3.9^b^	3.6^b^
**Health**						
Nonsmoker	11.1	9.6	9.8	10.3	10.9	10.4
Smoker	7.6	7.8	8.1	8.9	9.5	8.4
* Difference in mean expenditure share*^a^	3.5^b^	1.8^b^	1.7^b^	1.4^b^	1.4^c^	2.0^b^
**Transport**						
Nonsmoker	3.5	4.4	4.6	5.4	7.0	4.9
Smoker	3.6	3.8	4.4	5.0	7.1	4.9
* Difference in mean expenditure share*^a^	−0.1	0.6^b^	0.2	0.4^c^	−0.2	0.1
**Clothing and footwear**						
Nonsmoker	2.9	4.4	5.0	5.3	5.4	4.5
Smoker	3.2	3.8	4.4	4.8	5.2	4.3
* Difference in mean expenditure share*^a^	−0.3^c^	0.6^b^	0.6^b^	0.5^b^	0.2	0.1^c^
**Alcoholic beverages, tobacco and narcotics**						
Nonsmoker	0.0	0.0	0.0	0.0	0.0	0.0
Smoker	11.1	10.9	10.3	10.1	8.8	10.2
* Difference in mean expenditure share*^a^	−11.1^b^	−10.9^b^	−10.3^b^	−10.1^b^	−8.8^b^	−10.2^b^
**Restaurants and hotels**						
Nonsmoker	3.6	3.9	4.1	4.2	4.3	4.0
Smoker	4.0	4.2	4.3	4.4	4.6	4.3
* Difference in mean expenditure share*^a^	−0.5^c^	−0.3^c^	−0.2	−0.2	−0.3	−0.3^b^
**Education**						
Nonsmoker	1.2	2.5	3.7	4.3	6.1	3.4
Smoker	1.2	2.0	2.9	3.4	4.6	2.9
* Difference in mean expenditure share*^a^	−0.1	0.5^c^	0.8^b^	0.9^b^	1.5^b^	0.5^b^
**Furnishings, household equipment and maintenance**						
Nonsmoker	3.9	4.1	3.9	3.9	3.8	3.9
Smoker	3.6	3.6	3.4	3.5	3.4	3.5
* Difference in mean expenditure share*^a^	0.3^b^	0.5^b^	0.5^b^	0.5^b^	0.3^c^	0.4^b^
**Communication**						
Nonsmoker	1.6	2.1	2.3	2.6	2.9	2.3
Smoker	1.6	1.9	2.0	2.2	2.7	2.1
* Difference in mean expenditure share*^a^	0.0	0.2^b^	0.4^b^	0.4^b^	0.3^b^	0.2^b^
**Recreation and culture**						
Nonsmoker	0.8	1.4	1.6	1.7	2.7	1.6
Smoker	0.7	0.9	1.1	1.4	2.0	1.3
* Difference in mean expenditure share*^a^	0.0	0.5^b^	0.5^b^	0.3^c^	0.7^c^	0.3^b^
**Miscellaneous goods and services**						
Nonsmoker	3.2	3.9	4.0	4.0	4.1	3.8
Smoker	3.7	3.8	3.8	4.0	3.9	3.8
* Difference in mean expenditure share*^a^	−0.5^b^	0.1	0.2	0.1	0.1	−0.1

Positive values indicate that the expenditure on this category by nonsmoker households is higher than the expenditure of smoker households

a Difference in mean expenditure share between nonsmoker and smoker households. ANOVA, showing between all quintile groups (combined) p-value. Pairwise comparisons were done by independent samples t-test

b The difference is statistically significant at the 1% level

c The difference is statistically significant at the 5% level

Cigarette smoker households spent a significantly smaller proportion of their budget than waterpipe tobacco smokers on food and nonalcoholic beverages (−4.6%) (*p* = 0.009) and spent more on tobacco (4.3%) (*p* < 0.001) across all quintiles. In cigarette smoker households, the 1st (poorest) quintile spent a significantly larger proportion of their budget than the 5th (richest) quintile on food and nonalcoholic beverages (7.1%), housing (3.0%), and tobacco (3.3%), but spent a significantly lower proportion of their budget on health (−1.9%), and education (−3.3%) (*p* < 0.001). In waterpipe tobacco smoker households, the 1st quintile spent a significantly larger proportion of their budget than the 5th quintile on food and nonalcoholic beverages (9.8%), and housing (4.0%), but spent a significantly lower proportion of their budget on transport (−4.0%), and education (−1.9%) (*p* < 0.001). Waterpipe tobacco smoker households’ spending on tobacco (any smoked type) did not vary much across the 5 quintiles (Table 5). However, after excluding dual use in waterpipe smoker households, the mean expenditure on waterpipe tobacco was highest in the poorest quintile (5.2%, 4.5%, 3.5%, 3.6%, 4.0%, in the 1st, 2nd, 3rd, 4th, 5th quintiles respectively) (data not shown).

**Table 5:** Average annual household expenditure share and difference in mean expenditure share (%) between cigarette and waterpipe tobacco smoker households by income quintile, HIECs 2017/2018, Egypt

Expenditure share (%)	Income quintiles					Total
	1st (poorest)	2nd	3rd	4th	5th (richest)	
**Food and nonalcoholic beverages**						
Cigarette smoker households	39.5	38.4	37.9	36.2	32.4	36.7^b^
Waterpipe tobacco smoker households	44.2	44.2	41.0	40.6	34.4	41.3^b^
Difference in mean expenditure share^a^	−4.2	−5.8	−3.1	−4.4	−2.0	−4.6
**Housing, water, electricity, gas and other fuel**						
Cigarette smoker households	18.4	18.1	17.1	15.8	15.4	16.8^b^
Waterpipe tobacco smoker households	20.4	19.1	17.3	16.2	16.4	18.0^b^
* Difference in mean expenditure share*^a^	−2.0	−1.0	−0.2	−0.3	−0.9	−1.2
**Health**						
Cigarette smoker households	7.5	7.8	7.9	8.8	9.4	8.3^b^
Waterpipe tobacco smoker households	8.0	7.5	9.8	8.8	11.1	8.9
* Difference in mean expenditure share*^a^	−0.5	0.3	−1.8	0.0	−1.7	−0.6
**Transport**						
Cigarette smoker households	3.8	3.9	4.4	5.0	7.2	4.9
Waterpipe tobacco smoker households	3.3	3.4	4.1	4.6	7.3	4.3^b^
* Difference in mean expenditure share*^a^	0.5	0.5	0.3	0.4	−0.1	0.6
**Clothing and footwear**						
Cigarette smoker households	3.2	3.8	4.3	4.8	5.2	4.4^c^
Waterpipe tobacco smoker households	2.9	4.2	4.3	4.7	5.1	4.2^c^
* Difference in mean expenditure share*^a^	0.3	−0.4	0.0	0.1	0.1	0.2
**Alcoholic beverages, tobacco and narcotics**						
Cigarette smoker households^d^	12.6	11.8	11.0	10.7	9.3	10.9^b^
Waterpipe tobacco smoker households^d^	7.0	6.4	6.6	6.7	6.8	6.7^b^
* Difference in mean expenditure share*^a^	5.6	5.4	4.5	4.0	2.4	4.3
**Restaurants and hotels**						
Cigarette smoker households	4.2	4.2	4.3	4.4	4.6	4.4^b^
Waterpipe tobacco smoker households	3.5	3.8	3.8	4.0	3.7	3.8^c^
* Difference in mean expenditure share*^a^	0.7	0.4	0.6	0.4	0.9	0.6
**Education**						
Cigarette smoker households	1.3	2.0	2.8	3.4	4.6	3.0^b^
Waterpipe tobacco smoker households	1.0	1.5	3.2	2.5	2.9	2.2^b^
* Difference in mean expenditure share*^a^	0.3	0.5	−0.4	0.9	1.7	0.8
**Furnishings, household equipment and maintenance**						
Cigarette smoker households	3.5	3.6	3.3	3.4	3.4	3.4^b^
Waterpipe tobacco smoker households	3.9	3.7	3.3	4.0	3.5	3.7
* Difference in mean expenditure share*^a^	−0.4	−0.1	0.1	−0.6	−0.1	−0.3
**Communication**						
Cigarette smoker households	1.6	1.9	2.0	2.3	2.7	2.1^b^
Waterpipe tobacco smoker households	1.5	1.7	1.8	2.1	2.1	1.8^b^
* Difference in mean expenditure share*^a^	0.1	0.2	0.2	0.2	0.6	0.3
**Recreation and culture**						
Cigarette smoker households	0.7	0.9	1.1	1.3	2.0	1.2^b^
Waterpipe tobacco smoker households	0.7	0.8	1.1	1.6	2.1	1.2
* Difference in mean expenditure share*^a^	0.0	0.1	0.0	−0.3	−0.1	0.0
**Miscellaneous goods and services**						
Cigarette smoker households	3.7	3.7	3.8	4.0	3.9	3.8
Waterpipe tobacco smoker households	3.6	3.8	3.7	4.3	4.5	3.9
* Difference in mean expenditure share*^a^	0.1	−0.1	0.1	−0.3	−0.6	−0.1

Positive values indicate that the expenditure on this category by cigarette smoker households is higher than the expenditure of waterpipe tobacco smoker households

a Difference in mean expenditure share between cigarette and waterpipe tobacco smoker households. ANOVA with Bonferroni correction, showing between all quintile groups (combined) p-value. Pairwise comparisons were done by independent samples t-test

b The difference is statistically significant at the 1% level

c The difference is statistically significant at the 5% level

d Expenditure includes all types of smoked tobacco in the household

### Factors associated with household expenditure on cigarette and waterpipe tobacco

In the generalized linear model, higher household expenditure on cigarettes was independently associated with the head of household being male (*p* < 0.001), of older age (*p* = 0.003), unmarried (*p* < 0.001), employed part-time (*p* = 0.002), having a lower education level (*p* < 0.001), and not having chronic diseases (*p* = 0.013), and with urban residence (*p* = 0.011) and lower income (*p* < 0.001). Higher household expenditure on waterpipe tobacco was independently associated with urban households (*p* = 0.015), and lower income households (*p* < 0.001) **(**Table 6**)**.

**Table 6:** Generalized linear models of factors associated with annual household tobacco expenditure, HIECs 2017/2018, Egypt

Head of household is/has…	Annual household tobacco expenditure							
	Cigarettes				Waterpipe tobacco			
	β	SE	Wald Chi-Square	*p*-value	β	SE	Wald Chi-Square	*p*-value
**Intercept**	8.951	0.041	47144.691	< 0.001	8.131	0.182	1988.647	< 0.001
**Gender (male)**	0.208	0.045	21.609	< 0.001	−0.046	0.200	0.052	0.820
**Gender (female)**	**Reference**							
**Age (≥ 50 years old)**	0.060	0.020	8.843	0.003	0.002	0.091	0.001	0.982
**Age (< 50 years old)**	Reference							
**Residence (urban)**	0.046	0.018	6.461	0.011	0.222	0.091	5.958	0.015
**Residence (rural)**	**Reference**							
**Education (university or higher)**	−0.191	0.031	38.149	< 0.001	−0.298	0.177	2.854	0.091
**Education (secondary)**	−0.160	0.052	9.327	0.002	−0.402	0.278	2.103	0.147
**Education (intermediate)**	−0.093	0.021	19.386	< 0.001	−0.025	0.104	0.059	0.808
**Education (primary or less)**	**Reference**							
**Employment (full-time)**	0.012	0.026	0.224	0.636	0.013	0.122	0.012	0.913
**Employment (part-time)**	0.105	0.033	9.845	0.002	0.010	0.152	0.004	0.949
**Employment (unemployed)**	Reference							
**Marital status (married)**	−0.246	0.344	51.240	< 0.001	−0.189	0.162	1.353	0.245
**Marital status (unmarried)**	Reference							
**History of chronic diseases (yes)**	−0.053	0.021	6.158	0.013	−0.087	0.101	0.739	0.390
**History of chronic diseases (no)**	**Reference**							
**Income quintile (1st**,** poorest)** (5802.0 to 33280.0 EGP)	−0.729	0.0402	329.674	< 0.001	−0.719	0.129	31.067	< 0.001
**Income quintile (2nd )** (33286.0 to 43739.0 EGP)	−0.530	0.0295	322.023	< 0.001	−0.606	0.123	24.346	< 0.001
**Income quintile (3rd )** (43750.0 to 55035.0 EGP)	−0.388	0.0259	223.599	< 0.001	−0.715	0.126	32.184	< 0.001
**Income quintile (4th )** (55055.0 to 72500.0 EGP)	−0.234	0.0233	100.832	< 0.001	−0.442	0.112	15.643	< 0.001
**Income quintile (5th, richest)** (72511.5 to 7480000.0 EGP)	**Reference**							

## Discussion

Our analysis of data from 12,845 households in the HIECs 2017/2018 revealed that the proportion of smoking households were almost half of the sample; two-fifths (41.1%) were cigarette and less than a tenth (7.0%) were waterpipe tobacco smoking households. While cigarette smoking prevalence was higher, waterpipe tobacco smoking prevalence was lower in Egypt than estimates from household surveys in neighboring countries, such as Jordan (32.0%, 11.0%), Lebanon (35.1%, 39.5%), and Palestine (28.2%, 12.9%), respectively [16]. This variation could be due to reporting household versus individual smoking prevalence or due to cultural differences, specifically in social acceptability and true reporting of female smoking, as well as to the extent of tobacco control implementation. Higher prevalence of adult smoking is associated with lower income [17]. However, to our knowledge, there are no published studies on how tobacco expenditure affects the Egyptian household welfare.

Our findings show that Egyptian smoker households allocated a significant portion, more than a tenth (10.2%) of their budget, to tobacco in 2017/2018. This proportion is on the higher end of previous reports: 5% in Iran [6] and Mexico [7], 9% in Ghana [5], and 11% in China and Zimbabwe [4]. In Egypt, expenditure on cigarettes was triple that on waterpipe tobacco. This may be explained by several factors. Waterpipe tobacco smoking is twice as affordable [8] than cigarettes and was associated in this sample with rural residence, where prices are much lower than in urban areas. Also, two-fifths of waterpipe tobacco smoker households lived below the national poverty line (compared to one-quarter of cigarette smoker households, which is similar to the total proportion of poverty in this sample). In Egypt, Awwad et al., 2020 reported that rural areas had higher deprivation indices for health and education expenditure groups and called for programs to alleviate economic vulnerability of these households [18].

In 2017/2018, Egyptian smoker households spent less than nonsmoker households on virtually all expenditure groups, diverting necessary funds that could have been alternatively allocated to other commodities, in a preliminary indication of potential compromises associated with tobacco expenditure [4]. Previous studies reported that tobacco user households spent less on items that are associated with improved household welfare [4–7]. Consistent with these findings, we found that in Egypt the tobacco expenditure share in the total household budget was nearly equal to shares for clothing and transport, but was higher than shares for education, furnishings and housing equipment and maintenance, communication, and recreation. In our study, the poorest spent more than a tenth of their household budget on tobacco, reaching 10 times as much as on other expenditure groups, such as education. By compromising expenditure on education, expenditure on tobacco adversely impacts generations by deterring their development and future earning potential, and contributing to a vicious circle of household impoverishment [4].

We further examined how spending on tobacco displaced expenditure on other expenditure groups by the type of tobacco smoked. Egyptian cigarette smoker households spent less on food and housing but more on tobacco than waterpipe tobacco smoker households. As our analysis showed that cigarette smoker households had generally higher income levels than waterpipe tobacco smoker households, it could be argued that cigarette smoker households in this sample suffered from secondary poverty, while waterpipe tobacco smokers suffered from primary poverty. Unlike households that do not have sufficient income to afford their basic needs (primary poverty), households that have sufficient resources and are otherwise above the national poverty line, but due to the compromising effect and inefficient use of resources, are unable to meet basic needs (secondary poverty) [17, 19]. This explains the differentials we found in background characteristics between cigarette and waterpipe tobacco smoker households. The common independent factors associated with higher household expenditure on both cigarettes and waterpipe tobacco were urban residence and lower income. Future quantitative and qualitative studies could examine a potential hypothesis on the association between the revealed socioeconomic patterns with smoking in different income groups: people belonging to some socioeconomic groups might consider waterpipe tobacco use a normal element of their lifestyle. Another slightly more affluent group might consider cigarette use to be a part of their normal behavioral pattern. The third group with an even higher income might believe that tobacco use is what poorer people do. The tobacco industry may have used this pattern in promoting cigarettes among the poorer population that was traditionally practicing waterpipe tobacco smoking.

**Fig. 3:**
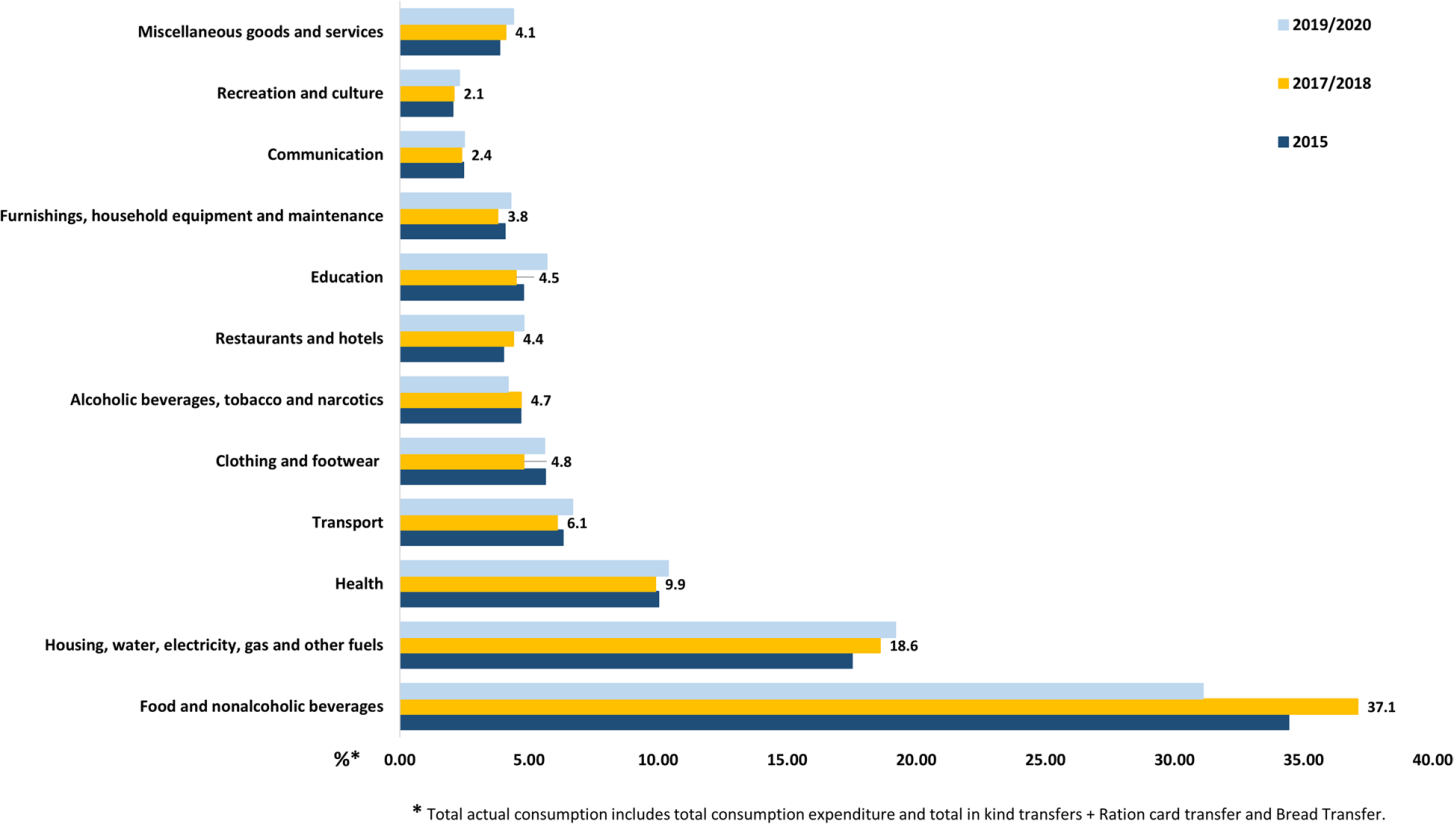
Expenditure share from the total household expenditure. Household Income, Expenditure and Consumption Surveys (2015–2020), Egypt

We argue that the sub-optimal implementation of the existing tobacco control interventions in Egypt [20], including tobacco taxation [21, 22], is behind the lack of a significant decline in the overall tobacco use prevalence seen over the last decade between 2012 and 2022 **(**Fig. 1**)**. In 2017/2018, there was a drop in the prevalence of tobacco use compared to the 2015 HIECs estimates that may be associated with the aftermath of the first devaluation of the EGP by 80% of its value. However, the minimal decline that continued into the COVID-19 pandemic era may be attributed, among other factors, to the government’s temporary stringent enforcement of smoke-free public places. Furthermore, there was nearly no change in mean annual household tobacco expenditure shares across the most recently published HIECs reports: 2015, 2017/2018, and 2019/2020 **(**Fig. 3**)**. Previous contextualized tax models reported a significant reduction in prevalence and premature deaths associated with cigarette [23] and waterpipe tobacco [22] smoking, but have not taken the distributional impacts into account [24]. These findings, taken together with our analysis of the 2017/2018 HIECs, offer evidence-based data on the Egyptian population’s consumption and expenditure behavior, especially pertaining to tobacco. Such information is vital to inform a more effective tobacco taxation policy. Nevertheless, only the optimal implementation of tobacco taxation within a comprehensive tobacco control framework could materialize the desired public and economic health benefits. In the WHO global report on trends in prevalence of tobacco use 2000–2025, Egypt was one of a handful of countries where tobacco use is still rising [25]. Also, the tobacco industry interference in Egypt is high [26]; the International Monetary Fund has instructed Egypt to sell off its tobacco monopoly, which it has sold to one of the global tobacco corporations [27, 28]. In the past, the end of monopolies and the advance of global tobacco corporations has led to higher smoking rates [29].

### Strengths of the study

We offer the first insights on how tobacco expenditure impacts the Egyptian household welfare using a large sample of national household data. To date, no published study has examined such impacts after the first EGP devaluation by 80% in 2016, and examined the differences by income quintile. In particular, this is the first attempt to compare between cigarette and waterpipe tobacco smoker household expenditures, as these are the two most used tobacco types in Egypt. Our analysis explored the socioeconomic inequalities and the effect of tobacco expenditure on household spending patterns in Egypt. We address a documented need from the policymaker perspective for characterization of household tobacco expenditure, conveyed during a previous joint policy brief and dialogue [22, 30–32]. This baseline profiling can potentially contribute to supporting the reduction of tobacco use associated socioeconomic inequalities. This study provides the baseline evidence required for modeling the health and economic benefits and the distributional effects of an improved tobacco taxation policy. We detailed the required model inputs for an improved tobacco taxation policy in our previous work [22, 32]. In this study, we provide contextualized baseline evidence regarding these model inputs, such as the national household expenditure on cigarette and waterpipe tobacco products and tobacco household expenditure share across income quintiles. These model inputs can contribute to developing different tobacco tax structure and burden scenarios that more efficiently reduce tobacco-related disparities. For instance, the existing ad valorem tax structure in Egypt is susceptible to undervaluation and can lead to lower prices of tobacco products. However, introducing specific excise tax can yield higher prices, particularly for low-priced products such as waterpipe tobacco [32]. Thus, researchers enable policymakers to select the most efficient taxation policy scenario that yields maximum health and economic benefits. Policymakers can then clearly examine which tax changes could be prioritized for adopting an improved tobacco taxation policy and how this change could affect the Egyptian household tobacco spending, taking the distributional impacts into account.

### Limitations of the study

There are some study limitations. Background data, including current smoking status, were available in the HIECs dataset used for this analysis at the level of heads of households only. Individual level data were not available. Data on tobacco expenditure were provided collectively with expenditure on alcohol and narcotics as one expenditure group. However, the household expenditure allocated to alcohol and narcotics was negligible. For household tobacco use and expenditure, we were able to obtain additional data on special request reflecting expenditure for all household smokers not just the head of household, as reported by the head of household not at the individual level. This limitation within the dataset did not allow analysis of the impact of expenditure decisions on the individual level. We focused in this analysis on cigarette and waterpipe tobacco, the most used tobacco products in Egypt. In this analysis, we did not include a separate category for dual smoker households (2.0%) or other smoked tobacco (< 1%) for simplification purposes. We presented the actual situation, where cigarette and waterpipe tobacco smoking households were not mutually exclusive. We examined differences between the poorest and richest in this sample using household income quintiles, as followed in previous studies [5, 7]. Other classifications have been used to distinguish poor from better-off households [6, 18]; these could be used in future in-depth studies. Finally, we did not use the current generation of econometric methods to estimate the crowding-out impact of tobacco expenditure as these entail more sophisticated statistical techniques, and each estimation method comes with a set of shortcomings [4]. However, for checking the differences in mean expenditure shares between smoker and nonsmoker households, simpler methods could be used [4]. The updated Toolkit on Using Household Expenditure Surveys for Research in the Economics of Tobacco Control (2023) recommended the two-sample t-test for equality of means for this purpose, noting that “it does not control for other household-specific characteristics that may influence budget allocation decisions” [4]. However, it is suitable for providing a “preliminary indication of potential compromises, if any, made as a result of tobacco spending” [4]. In addition, we used multivariable methods to detect differences in mean expenditure shares by income quintiles. Share-based estimates may be biased; thus, recognizing this limitation could be a first step towards reconsidering the existing and widely used approaches. Future studies with adequate funding can use more advanced econometric methods for estimating the crowding-out effect of tobacco expenditure.

## Conclusion

This study offers the first insights on how tobacco expenditure impacts the Egyptian household welfare, comparing between smoker and nonsmoker households, as well as cigarette and waterpipe tobacco households, by income quintiles. Our analysis explored the socioeconomic inequalities and the effect of tobacco expenditure on household spending patterns in Egypt. In 2017/2018, one tenth of the Egyptian smoker household’s expenditure was allocated to tobacco, disproportionately concentrated among the poorest. Smoker households spent less than nonsmoker households on virtually all expenditure groups, preliminarily indicating that tobacco spending is associated with potential compromises of varying extent in other expenditure groups in smoker households. Cigarette smoker households spent less on food and housing, but more on tobacco than waterpipe tobacco smoker households. The smoker household expenditure on cigarettes was triple that on waterpipe tobacco. More waterpipe tobacco than cigarette smoker households lived below the poverty line, suggesting the latter group was driven into secondary poverty due to tobacco expenditure. The poorest waterpipe tobacco and cigarette smoker households spent 7–10 times as much on tobacco as on education, reflecting the long-term adverse implications of tobacco expenditure on household impoverishment and development. This analysis provides the evidence-based characterization of household tobacco expenditure that is required for modeling the health and economic benefits and the distributional effects of an improved tobacco taxation policy. This baseline profiling can potentially contribute to a uniform tobacco taxation policy supporting the reduction of the tobacco use associated socioeconomic inequalities.

## Data Availability

No datasets were generated or analysed during the current study.
